# Special Issue: Pathophysiology and Therapeutic Perspectives in DMD: The Well-Defined Role of the Immune System

**DOI:** 10.3390/biomedicines9121911

**Published:** 2021-12-14

**Authors:** Andrea Farini

**Affiliations:** Laboratorio di Cellule Staminali, Dipartimento di Fisiopatologia Medico-Chirurgica e dei Trapianti, Università degli Studi di Milano, Fondazione IRCCS Cà Granda Ospedale Maggiore Policlinico, Milano, Centro Dino Ferrari, Via Francesco Sforza 35, 20122 Milan, Italy; farini.andrea@gmail.com

Duchenne muscular dystrophy (DMD) is the most common, lethal, muscle-wasting disease of childhood. Dystrophin absence alters the contraction machinery and, due to fiber degeneration/regeneration cycles, dystrophic muscles develop fibrosis as a consequence of continuous muscular injury and regenerative potential exhaustion. Together with the loss of sarcolemma stability and the increase in muscular weakness, this condition determines cardiac/respiratory failure and premature death. Although the primary defects rely on skeletal muscle structure, a multitude of secondary defects exist involving metabolic—as well as inflammatory—deregulated pathways. Indeed, immune cell infiltration into the skeletal muscle is a notable feature of the disease pathophysiology and is strongly associated with disease severity. 

Immunosuppressive drugs, such as glucocorticoids, are the only effective therapy to delay the onset and control symptoms in a number of patients, but they cause serious side adverse effects, limiting their use in patients. Consistently, a deeper understanding of inflammation-driven mechanisms is fundamental to produce new feasible treatments. In this Special Issue, we reviewed the latest studies on DMD pathogenesis to assess the complex relationship between innate and adaptive immune responses in dystrophic skeletal muscles [1] and open the way for the identification of new therapeutic targets. In particular, Tulangekar et al. described how neutrophils’ secretion of myeloperoxidase (MPO) and neutrophil elastase (NE) improved the rise in oxidative stress and consequently the development of inflammation, suggesting their modulation as feasible clinical outcomes [2]. Similarly, Herbelet and co-workers investigated the protein kinase A/mitogen-activated protein kinase/nuclear factor of the activated T-cells 5/organic osmolytes (PKA-p38MAPK-NFAT5-organic osmolytes) pathway and its role in mediating muscle homeostasis and skeletal muscle regeneration [3].

Regarding the advent of new therapeutic treatments to improve the condition of disabled patients, Testa et al. suggested that the cell-based reconstructive approach could be a feasible approach to circumvent chronic dysfunctions in healing processes that affect DMD muscles [4]. 

With the same effort to find the optimal muscle progenitor source that show both myogenic potential and high proliferation rates to obtain a sufficient amount of cells, Shen et al. demonstrated that activated mesenchymal stem cells (MSCs) improved the function of distal skeletal muscles following implantation in dystrophic animal models [5]. Similarly, Park et al. demonstrated that the injection of MSCs isolated from Wharton’s jelly in mdx mice resulted in the amelioration of a pathological phenotype, as they found the down-regulation of CK, apoptosis, and fibrosis, probably through the inhibition of miR-499-5p and its targets, TGFβR-1 and -3 [5].

Interestingly, Uchimura and Sakurai obtained differentiated myotubes from DMD patient-derived induced pluripotent stem cells (iPSCs) and showed that the modulation of the activity of calcium channels STIM1 and Orai1 rescued the contractile properties of dystrophic muscles [6]. Dubinin et al. studied the efficacy of alisporivir, a non-immunosuppressive inhibitor of mitochondrial permeability transition pore (MPTP), to modulate the cardiac phenotype of mdx mice, and they found that mitochondrial biogenesis was inhibited, and the amount of mtDNA was markedly reduced [7].

It is well accepted that the most efficacious therapy for DMD should attempt to block the immune responses that are provoked by the inflammatory milieu and preserve the reparative functions in myogenesis that are normally exerted by inflammation. At the same time, other therapeutic interventions are needed to rescue the effects of non-inflammatory dysfunctions, such as those related to mitochondria or calcium influx, that dramatically worsen myofibers’ degeneration. 

In this regard, in this Special Issue, it was suggested that early and precise combined interventions with regard to inflammatory pathways that affect the skeletal muscle of DMD patients are mandatory both to enhance the feasibility of gene therapy and to allow safer cellular transplantation.
